# Two-hour post-challenge hyperglycemia, but not fasting plasma glucose, associated with severity of coronary artery disease in patients with angina

**DOI:** 10.1371/journal.pone.0202280

**Published:** 2018-08-15

**Authors:** Chia-Po Fu, Wayne H-H Sheu, Wen-Lieng Lee, Wen-Jane Lee, Jun-Sing Wang, Shih-Yi Lin, I-Te Lee

**Affiliations:** 1 Division of Endocrinology and Metabolism, Department of Medicine, Taichung Veterans General Hospital, Taichung, Taiwan; 2 Graduate Institute of Biomedical Electronics and Bioinformatics, College of Electrical Engineering and Computer Science, National Taiwan University, Taipei, Taiwan; 3 Institute of Biomedical Science, Chung Sing University, Taichung, Taiwan; 4 Department of Medicine, National Defense Medical Center, Taipei, Taiwan; 5 School of Medicine, National Yang Ming University, Taipei, Taiwan; 6 Cardiovascular Center, Taichung Veterans General Hospital, Taichung, Taiwan; 7 Department of Medical Research, Taichung Veterans General Hospital, Taichung, Taiwan; 8 Tung-Hai University, Taichung, Taiwan; 9 Center for Geriatrics and Gerontology, Taichung Veterans General Hospital, Taichung, Taiwan; 10 School of Medicine, Chung Shan Medical University, Taichung, Taiwan; Osaka University Graduate School of Medicine, JAPAN

## Abstract

**Introduction:**

Postprandial hyperglycemia plays a pivotal role in cardiovascular disease. However, few studies have investigated associations between the severity of coronary artery disease (CAD) and postprandial glucose levels in angina patients without known diabetes before coronary angiography.

**Methods:**

Subjects who were admitted for coronary angiography due to angina and were in stable condition after discharge were recruited. A standard 75-g oral glucose tolerance test (OGTT) was performed at outpatient visits approximately 2–4 weeks after hospital discharge, and fasting and post-challenge blood glucose were measured. Twenty-six volunteers in our hospital staff served as the healthy group. CAD severity was graded using the SYNTAX and Jeopardy scoring systems.

**Results:**

The subjects in the angina group had a higher body mass index, higher fasting glucose, and higher 2-h postprandial glucose than those in the healthy group. The SYNTAX and Jeopardy scores were significantly associated with 2-h postprandial blood glucose (correlation coefficients = 0.164 and 0.187, respectively) but not with fasting glucose. Linear regression analyses revealed that SYNTAX and Jeopardy scores were independently associated with glucose levels at 120 min after OGTT (SYNTAX 95%CI = 0.003–0.103; Jeopardy score 0.002–0.027) but not with fasting glucose.

**Conclusion:**

CAD severity is associated with blood glucose levels after oral glucose challenge in patients without known diabetes before coronary angiography, suggesting that CAD patients should be routinely screened for post-challenge blood glucose.

## Introduction

The incidence of type 2 diabetes mellitus (DM) is gradually increasing worldwide. Studies predict that in 2040, one in ten adults will have DM, and one in every two adults with DM will go undiagnosed [1]. Patients with DM are at a high risk of micro- and macro-vascular disease, and this risk can be reduced through rigorous blood glucose control and lowering of hemoglobin A1c (HbA1c) [2,3]. Pre-diabetes refers to impaired fasting glucose and impaired glucose tolerance, which is a metabolic state between normal glucose metabolism and DM. It is an important risk factor for DM and coronary vascular disease [4]. Furthermore, the severity of coronary artery disease is more strongly associated with pre-diabetes status than with normal glucose metabolism [5]. Many previous studies have shown that fasting plasma glucose (FPG) and HbA1c are associated with the risk of cardiovascular disease. However, an increasing number of emerging studies have determined that post-prandial glucose (PPG) plays a vital role in cardiovascular disease [6–9]. Fewer studies have reported associations between the severity of CAD and FPG, PPG and HbA1c.

The SYNTAX (Synergy between PCI with Taxus and Cardiac Surgery) score is a comprehensive angiographic severity scoring system that quantifies CAD complexity [10,11]. The Jeopardy score is another measure of severity that is simpler to calculate than the SYNTAX score; it is designed to estimate whether the myocardium is in jeopardy [12]. In the present study, we aimed to clarify the associations between the severity of CAD (measured by the SYNTAX and Jeopardy scores) and FPG, PPG and HbA1c in angina patients without known diabetes before coronary angiography.

## Methods

### Study participants

Patients aged 20 years and older without history of DM who had been admitted for coronary artery angiography (CAG) due to angina were enrolled [13]. The severity of CAD was measured using SYNTAX score and Jeopardy score calculations, which were calculated by one cardiologist who was blinded to the patients’ biochemistry data. A standard 75-g oral glucose tolerance test (OGTT) was performed at outpatient visits approximately 2–4 weeks after hospital discharge, and blood samples were collected to detect glucose levels at fasting and 120 minutes after glucose challenge. In addition, 26 male volunteers with normal weight and without CAD in our hospital staff served as a healthy group. The study was approved by the Institutional Review Board of Taichung Veterans General Hospital, Taichung, Taiwan, and all study subjects provided written informed consent before the study procedure began.

### Biochemical analysis

Blood glucose, lipids, liver enzymes, and creatinine were measured after an overnight fast at the central clinical laboratory of the hospital by enzymatic methods using a chemistry analyzer (Hitachi 7600, Hitachi Co., Tokyo, Japan). High-sensitivity C-reactive protein (Hs-CRP) was determined using an immunochemical assay (Good Biotech Corp.). The mean intra- and inter-assay CVs for hs-CRP were 1.4% and 1.4%, respectively.

### Statistical analysis

All data are expressed as the mean±SD. Student’s *t*-test was used for comparisons between groups. Fisher’s exact test was applied for gender. Pearson’s correlation test was performed to examine the relationships between continuous variables and the SYNTAX and Jeopardy scores. The non-parametric Mann-Whitney test was used to analyze glutamic pyruvic transaminase (GPT) and hs-CRP since they are not normally distributed. In addition, multiple linear regression analyses were performed to determine associations between severity scores and OGTT, with SYNTAX or Jeopardy scores as the dependent variable and components of metabolic syndrome as the independent variables. The results were considered statistically significant at *P*<0.05. All data were analyzed using SPSS (Statistical Package for the Social Sciences) 18.0 for Windows (SPSS, Inc., Chicago, IL, USA).

## Results

A total of 240 subjects undergoing coronary angiography for angina were enrolled in this study. Compared with the healthy men, the angina patients were older and had a greater waist circumference, higher blood pressure, higher fasting glucose before the 75-g oral glucose challenge test (OGTT0’), higher glucose at 120 minutes after the 75-g oral glucose challenge test (OGTT120’). They also had higher serum creatinine, but no significant differences were found in GPT, total cholesterol or hs-CRP (Table 1). Low-density lipoprotein was lower in the angina group with 52.5% statin medication usage (Table 1). Using bivariate Pearson’s analysis, the SYNTAX score was positively correlated with the 2-h OGTT120’ (*r* = 0.164, *P*<0.05) and serum creatinine but not with systolic blood pressure, high-density lipoprotein (HDL) cholesterol, triglycerides, waist circumference or OGTT0’ (Table 2). The Jeopardy score was also positively associated with OGTT120’(*r* = 0.187, *P*<0.01) but not with OGTT0’ (Table 2). The SYNTAX score and Jeopardy score were higher in patients with 2-h glucose ≧140 mg/dl than in those with 2-h glucose <140 mg/dl (12.0±16.2 vs. 8.1±14.7, P<0.05 and 3.8±4.0 vs. 2.8±3.8, P<0.05), but neither of the severity scores were significantly different between subjects with fasting glucose ≧100 mg/dl and those with <100 mg/dl (9.7±15.1 vs. 10.2±15.8, *P* = NS and 3.4±3.9 vs. 3.3±3.9, *P* = NS). Using multiple linear regression analysis, SYNTAX and Jeopardy scores were independently associated with 2-h OGTT after adjustment for other confounding factors (Tables 3 and 4).

**Table 1:** Demographic and clinical data for the subjects with angina and healthy men.

	Angina	Healthy men	P value
	(n = 240)	(n = 26)	
Age	60±12	39±10	< 0.001
Gender (M/F)	197/43	26/0	0.011
BMI (kg/m^2^)	26.2±3.8	22.4±1.5	< 0.001
WC (cm)	91.0±9.1	82.0±6.2	< 0.001
SBP (mmHg)	127±18	114±10	< 0.001
DBP (mmHg)	74±10	68±7	0.015
Total cholesterol (mmol/L)	4.61±0.98	4.84±0.83	0.291
Triglycerides (mmol/L)	1.57±0.89	1.08±0.36	< 0.001
HDL cholesterol (mmol/L)	1.22±0.26	1.42±0.31	< 0.001
LDL cholesterol (mmol/L)	2.62±0.78	3.03±0.76	0.015
OGTT0’ (mmol/L)	5.33±0.78	4.99±0.39	0.039
OGTT120’ (mmol/L)	8.10±2.49	6.38±1.83	< 0.001
HbA1c (%)	5.9±0.5		
GPT (U/L)*	31±36	25±12	0.383
Creatinine (umol/L)	85.7±25.6	78.6±10.6	0.006
Hs-CRP (mg/L)*	1.82±1.96	1.81±4.2	0.962
SYNTAX score	10.0±15.6		
Jeopardy score	3.3±3.9		
Medications			
Antiplatelet, %	95		
ACEI/ARB, %	52.1		
β-blockers, %	28.3		
CCB, %	50.8		
Statins, %	52.5		
Family history			
DM (father), n	25		
DM (mother), n	45		
CAD (father), n	38		
CAD (mother), n	34		

BMI: body mass index; WC: waist circumference; SBP: systolic blood pressure; DBP: diastolic blood pressure; HDL: high-density lipoprotein; LDL: low-density lipoprotein; OGTT 0’: glucose before 75-g oral glucose challenge test; OGTT 120’: glucose at 120 minutes after 75-g oral glucose challenge test; GPT: glutamic pyruvic transaminase; HbA1c: hemoglobin A1c; Hs-CRP: high-sensitivity C-reactive protein; ACEI: angiotensin-converting enzyme; ARB: angiotensin II receptor antagonist; CCB: calcium channel blocker; DM: diabetes mellitus; CAD: coronary artery disease

*P-value between groups based on the non-parametric Mann-Whitney test.

**Table 2:** Subjects’ (N = 240) cardiovascular risk factors, evaluated by bivariate Pearson’s correlation coefficients (r).

	Age	BMI	WC	SBP	TC	HDL	TG	OGTT0	OGTT120	Cr	GPT	SYNTAX	Jeopardy
BMI	-0.245**												
WC	-0.11**	0.828**											
Systolic BP	0.271**	0.269**	0.241**										
Total cholesterol	-0.205**	-0019	-0.156*	0.003									
HDL cholesterol	-0.004	-0.217**	-0.318**	-0.033	0.392**								
TG	-0.188**	0.238**	0.248**	0.061	0.218**	-0.224**							
OGTT0’	0.084	0.107	0.202**	0.092	-0.083	-0.168**	0.059						
OGTT120’	0.326**	0.06	0.136*	0.247**	-0.097	-0.079	0.008	0.283**					
Creatinine	0.254**	-0.079	0.064	0.165*	-0.179**	-0.288**	0.026	0.205**	0.152**				
GPT	-0.153*	0.082	0.066	-0.074	0.210	-0.102	0.052	0.037	-0.009	-0.046			
SYNTAX	0.051	-0.034	0.029	0.016	0.011	-0.093	-0.046	0.065	0.164*	0.130*	0.062		
Jeopardy	0.043	0.015	0.091	0.066	-0.132	-0.129	-0.029	0.099	0.187**	0.183**	0.081	0.730**	
Hs-CRP	0.126	-0.069	0.028	0.084	0.035	-0.171**	-0.011	0.088	0.184**	0.259**	0.144	0.141*	0.118

*P<0.05.

**P<0.01.

BMI: body mass index; WC: waist circumference; BP: blood pressure; HDL: high-density lipoprotein; OGTT0’: glucose before 75-g oral glucose challenge test; OGTT120’: glucose at 120 minute after 75-g oral glucose challenge test; GPT: glutamic pyruvic transaminase; Hs-CRP: high-sensitivity C-reactive protein

**Table 3:** Multiple linear regression analysis of associations between SYNTAX score and 2-h OGTT in angina patients.

Independent variable	Multiple regression coefficient		*P* value	95% CI Of β	
	β	SE (β)		Lower limit	Upper limit
Age	-0.07	0.10	NS	-0.271	0.121
WC	0.26	0.22	NS	-0.167	0.684
BMI	-0.81	0.53	NS	-1.857	0.238
SBP	-0.02	0.06	NS	-0.146	0.104
OGTT 0’	-0.02	0.08	NS	-0.177	0.127
OGTT120’	0.05	0.03	0.038	0.003	0.103
Total cholesterol	0.06	0.03	NS	-0.006	0.122
HDL cholesterol	-0.21	0.13	NS	-0.459	0.039
Triglyceride	-0.02	0.01	NS	-0.049	0.007
Creatinine	4.25	3.84	NS	-3.317	11.824
Hs-CRP	0.39	0.54	NS	-0.670	1.442

CI: confidence interval; WC: waist circumference; BMI: body mass index; SBP: systolic blood pressure; OGTT 0’: glucose before 75-g oral glucose challenge test; OGTT 120’: glucose at 120 minutes after 75-g oral glucose challenge test; HDL: high-density lipoprotein; Hs-CRP: high sensitivity C-reactive protein

**Table 4:** Multiple linear regression analysis of associations between Jeopardy score and 2-h OGTT in angina patients.

Independent variable	Multiple regression coefficient		*P* value	95% CI Of β	
	β	SE (β)		Lowe limit	Upper limit
Age	-0.04	0.03	NS	-0.087	0.011
WC	0.06	0.05	NS	-0.042	0.171
BMI	-0.17	0.13	NS	-0.433	0.091
SBP	0.01	0.02	NS	-0.026	0.037
OGTT 0’	0.00	0.02	NS	-0.039	0.037
OGTT120’	0.01	0.01	0.026	0.002	0.027
Total cholesterol	-0.01	0.01	NS	-0.021	0.011
HDL cholesterol	-0.02	0.03	NS	-0.085	0.040
Triglyceride	-0.01	0.01	NS	-0.010	0.004
Creatinine	1.71	0.96	NS	-0.182	3.603
Hs-CRP	0.07	0.13	NS	-0.194	0.333

CI: confidence interval; WC: waist circumference; BMI: body mass index; SBP: systolic blood pressure; OGTT 0’: glucose before 75-g oral glucose challenge test; OGTT 120’: glucose at 120 minutes after 75-g oral glucose challenge test; HDL: high-density lipoprotein; Hs-CRP: high sensitivity C-reactive protein.

## Discussion

Our main finding is that 2-hour glucose but not fasting glucose was significantly associated with the severity of CAD as expressed by SYNTAX score or Jeopardy score in patients with angina.

Blood glucose levels fluctuate within a certain range in healthy subjects. The results of the present study indicate that postprandial glucose contributes to the progression of CAD in patients with angina. The post-challenge blood glucose level is a risk factor for CAD in subjects without diabetes [14], and daily glucose excursion affects coronary plaque vulnerability in patients with CAD and those pre-treated with lipid-lowering therapy [15]. The mechanism by which blood glucose excursion appears to reflect CAD severity may be associated with inflammation [16–19]. The RIAD study demonstrated that the pathophysiological basis of impaired fasting glucose (IFG) and impaired glucose tolerance (IGT) are different [20]. Subjects with IGT have higher levels of free fatty acids [20], which may predict the severity of myocardial ischemia [21], and this finding is consistent with our findings that patients with 2-h post-challenge glucose levels greater than 140 mg/dl had higher CAD severity scores but not higher fasting glucose levels. In the regression analysis, the association between post-challenge glucose and SYNTAX or Jeopardy scores remained after adjustment for the other CAD risk factors (age, waist circumference, BMI, systolic blood pressure, fasting glucose, total cholesterol, HDL cholesterol, triglyceride and creatinine). This indicated that in patients with angina but without known DM, both post-challenge glucose excursion and post-challenge blood glucose were higher compared with the healthy controls. In the present study, 23% of patients were newly diagnosed with DM, and the average HbA1c was 5.9%, suggesting that at lower HbA1c levels, the contribution of glucose variability becomes predominant. The contribution of postprandial glucose was greater in patients with lower HbA1c, and this occurs more often in Asian populations [22]. Patients in the present study who had post-challenge hyperglycemia according to 75-g OGTT results showed significant associations with the SYNTAX score, which was consistent with the results of Watanabe et al [23]. Moreover, the SYNTAX score was closely associated with the Jeopardy score (r = 0.73, *P*<0.001), suggesting that both the SYNTAX and Jeopardy scoring systems have a similar ability to estimate the extent and severity of CAD.

The results of a recent study showed that diurnal glycemic fluctuation is associated with the severity of CAD in *prediabetic* patients [23]. We enrolled individuals with angina but without diabetes prior to coronary angiography. Another recent study revealed that post-challenge insulin concentration but not HbA1c, fasting glucose, insulin, or post-challenge blood glucose level can differentiate between CAD and cardiac syndrome X in subjects without known diabetes [24]. Combined with the results of the present study, this implies that insulin and glucose play a pivotal role in the post-challenge blood test.

Obesity is a recognized independent risk factor for cardiovascular disease [25–28]. However, the obesity paradox (i.e., lower mortality in subjects who are overweight or obese) does not exist in subjects with or without diabetes [25]. Other studies have reported that clinical outcomes are improved in overweight and obese patients with diabetes [29], hypertension [30] and end-stage renal disease [31]. The present study demonstrated that obesity itself and its associated components (i.e., waist circumference, FPG, HDL-C, triglyceride or SBP) were not associated with SYNTAX or Jeopardy scores after adjustments for other confounding factors, which is consistent with a recent study that failed to detect associations between BMI and Jeopardy scores [32].

Our study has several limitations. First, this is a cross-sectional study, which limits inferences of causality, and a prospective study must be conducted to corroborate the results. Second, this study was conducted at a single medical center in central Taiwan within a specific time frame; thus, selection bias cannot be excluded. Third, because the study subjects needed to agree to undergo an OGTT 2–3 weeks after discharge, those who agreed may be more motivated and may have a higher self-care capability. Fourth, the subjects for comparison are not matched controls but healthy men. Finally, we only enrolled Asian subjects, so the results cannot be generalized to Caucasian populations, which may have a different core pathophysiology [33].

## Conclusions

In conclusion, the results of this study show that post-challenge blood glucose levels are associated with CAD severity in patients who have undergone coronary angiography due to angina. Our results suggest that postprandial glucose or post-challenge blood glucose should be routinely screened in patients diagnosed with CAD.

## Supporting information

S1 Dataset All the datasets collected can be freely downloaded with personal information removed. (RAR)
